# A Drastic Reduction in the Life Span of Cystatin C L68Q Carriers Due to Life-Style Changes during the Last Two Centuries

**DOI:** 10.1371/journal.pgen.1000099

**Published:** 2008-06-20

**Authors:** Astridur Palsdottir, Agnar Helgason, Snaebjorn Palsson, Hans Tomas Bjornsson, Birkir Thor Bragason, Solveig Gretarsdottir, Unnur Thorsteinsdottir, Elias Olafsson, Kari Stefansson

**Affiliations:** 1 Institute for Experimental Pathology, University of Iceland, Keldur, Reykjavik, Iceland; 2 deCODE Genetics, Reykjavik, Iceland; 3 Department of Anthropology, University of Iceland, Oddi, Reykjavik, Iceland; 4 Institute of Biology, University of Iceland, Askja, Reykjavik, Iceland; 5 Johns Hopkins University School of Medicine, McKusick-Nathans Institute of Genetic Medicine, Department of Pediatrics, Baltimore, Maryland, United States of America; 6 Faculty of Medicine, University of Iceland, Laeknagardur, Reykjavik, Iceland; 7 Department of Neurology, LSH University Hospital, Fossvogi, Reykjavik, Iceland; The Wellcome Trust Sanger Institute, United Kingdom

## Abstract

Hereditary cystatin C amyloid angiopathy (HCCAA) is an autosomal dominant disease with high penetrance, manifest by brain hemorrhages in young normotensive adults. In Iceland, this condition is caused by the L68Q mutation in the cystatin C gene, with contemporary carriers reaching an average age of only 30 years. Here, we report, based both on linkage disequilibrium and genealogical evidence, that all known copies of this mutation derive from a common ancestor born roughly 18 generations ago. Intriguingly, the genealogies reveal that obligate L68Q carriers born 1825 to 1900 experienced a drastic reduction in life span, from 65 years to the present-day average. At the same time, a parent-of-origin effect emerged, whereby maternal inheritance of the mutation was associated with a 9 year reduction in life span relative to paternal inheritance. As these trends can be observed in several different extended families, many generations after the mutational event, it seems likely that some environmental factor is responsible, perhaps linked to radical changes in the life-style of Icelanders during this period. A mutation with such radically different phenotypic effects in reaction to normal variation in human life-style not only opens the possibility of preventive strategies for HCCAA, but it may also provide novel insights into the complex relationship between genotype and environment in human disease.

## Author Summary

This work focuses on the L68Q mutation in the cystatin C gene which causes hereditary cystatin C amyloid angiopathy (HCCAA), an autosomal dominant disease with high penetrance in contemporary Icelanders, manifest by lethal brain hemorrhages in young normotensive adults. On the basis of both DNA haplotype and genealogical analyses, we determined that all known copies of the L68Q mutation derive from a common ancestor born roughly 18 generations ago, around 1550. Furthermore, we were able to show a rapid change in its penetrance for obligate carriers born from 1825 to 1900, demonstrated by a drastic reduction in life span, from 65 years to the present-day average of 30 years. The detrimental phenotypic impact of the L68Q mutation appears to have emerged in reaction to common life-style changes almost three centuries after the mutation occurred. We believe that this is the first report of phenotypic flexibility of a monogenic disease in reaction to life-style changes that fall within the normal range of behavior of a single population in the space of a few generations. Our results underline how single gene disorders with simple Mendelian inheritance can be affected by environmental factors, resulting in changed disease status.

## Introduction

Amyloid deposits are found in various diseases, both genetic and sporadic, such as Alzheimer's disease and the prionoses [1]. HCCAA (MIM #105150) has so far only been found in Iceland and it was the first to be described of a group of diseases called hereditary cerebral amyloid angiopathies, characterized by amyloid deposition in brain arteries [2],[3]. The cystatin C gene encodes an extracellular proteinase inhibitor with activity against cysteine proteases of the papain and legumain family [4]. The cystatin C L68Q mutation [5],[6] is highly penetrant and the disease is manifest by an intracerebral hemorrhage (ICH) in young normotensive adults. The ICH is recurrent if the patient survives the first attack and then commonly associated with dementia of variable severity and/or paralysis, leading to death at 30.7 years of age, on average (based on 130 individuals born after 1900 with life spans ranging from 15 to 79 years). Cystatin C is the predominant component [7] of the amyloid which is found in the cerebral arterioles of patients [8]. In addition, wild-type cystatin C is found in amyloid deposits with the Aβ peptide in Alzheimer's patients, modulating cerebral β-amylodosis [9]–[11]. Furthermore, the cystatin C locus (CST3) is one of the candidate susceptibility loci for sporadic Alzheimer's disease [12].

In the present study we collected data on patients diagnosed with HCCAA, traced their family trees and found a progressive reduction in the life span of patients and obigate carriers of the disease mutation in addition to a parent-of-origin effect.

## Results

### Families

**Figure 1:**
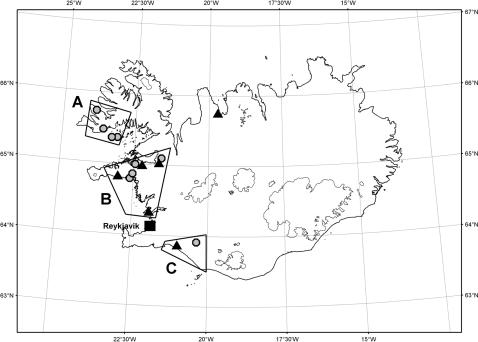
A map of Iceland demonstrating the geographical distribution of families with the HCCAA mutation around the year 1800. Families were distributed in three regions denoted A, B, and C. Circles indicate families from which individuals have been DNA diagnosed and triangles indicate families without DNA diagnosis.

**Figure 2:**
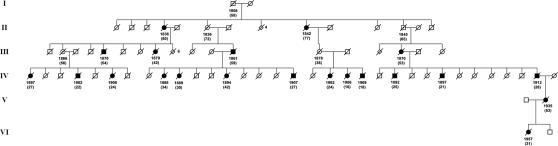
One of the HCCAA pedigrees. Women are depicted as circles, men as squares. The year of birth and life span (in brackets) are shown beneath the symbols. Triangles indicate children that died as infants; their number is indicated beside the triangle. Obligate mutation carriers are labeled with a central dot and diagnosed individuals (brain hemorrhage according to death certificates/parish records) are shown as filled symbols. As expected for a dominant disease, about half of the sib-groups are carriers on average. Within this pedigree only individuals V-II and VI-I were DNA diagnosed.

Overall, 15 families were identified with 266 known or inferred carriers of the L68Q mutation. Figure 1 depicts the geographical distribution of all 15 families around the year 1800 and Figure 2 shows an example pedigree for one extended family.

### Age of the Mutation

The mean uncorrected single marker estimate was 13.9 generations, which increased to 21.5 after applying the Luria-Delbrück correction for population growth rate (Table S1). Both means are affected by several extreme values obtained for microsatellites where founder alleles are still in very strong LD with the L68Q mutation.

An analysis of all 20 microsatellites simultaneously, using the DMLE+ software, yielded a single age estimate of 17.9 generations (95% C.I. 10.9–28.5). This suggests that the mutation occurred on a chromosome carried by an individual born around the mid 16^th^ century, presumably in Iceland.

### Earliest Ancestor

We could not find the common ancestor of all the families using genealogy due to lack of data. However, the earliest known common ancestor was a man, born in 1684 in region B, who moved and founded the two Southern families in region C (see Figure 1).

### Studies of Life Span

**Figure 3:**
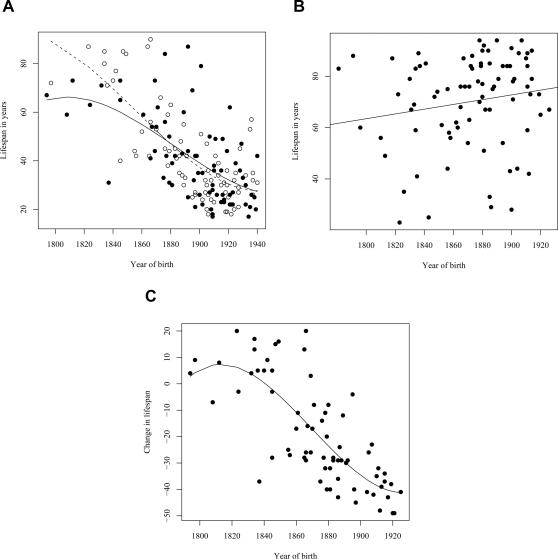
Families with DNA diagnosis. (A) Relationship between the life span of L68Q mutation carriers (n = 157) and their year of birth from the year 1800 to 1940. The observed values for females are denoted with open circles, males with filled circles. The expected values, from a polynomial regression, for females and males are shown with a dashed, and a solid line, respectively. The difference among the genders was not significant. (B) Relationship between the life span of 86 spouses of L68Q mutation carriers in families with DNA diagnosis and their year of birth. The line represents the fitted relationship from a linear regression (*b* = 0.093, *P*<0.1232, *R^2^* = 0.03). It should be noted that these individuals represent adults that reached child-bearing age; the figure, therefore, does not reflect the relatively high infant/child mortality rate of the period. (C) Difference in life-span of obligate gene carriers (n = 72), compared to the spouses group (n = 86), in relation to the birth year of the carriers. The line presents the expectation from a polynomial regression (*R^2^* = 0.59). The expectancy of life span during the study period was estimated by a regression of life span of spouses on their birth year (B). The reduction in life span of a carrier, considering his/her birth year, was calculated as the deviation from this line.

As premature death is such a striking phenotypic feature of the L68Q mutation in confirmed carriers, we next attempted to determine whether their ancestors carrying the mutation also had reduced life span. First, we examined only the 157 confirmed carriers, using their spouses as controls with the rationale that they originated from the same regions [13], show a similar distribution of birth years and led similar lifestyles as the mutation carriers. The dependence of life span on the year of birth was studied by linear regression models including polynomial terms. This analysis revealed that the life span of L68Q carriers, both men and women, underwent a significant reduction (*P*≤2.2*10^−16^) during the 19^th^ century (Figure 3A and Table 1). This life span reduction is evident in the pedigrees, an example of which is shown in Figure 2. In comparison, we found no change in the life span of the spouses (n = 84) during this period (*b* = 0.093, *P*≤0.1232, Figure 3B). The reduction in life span of L68Q carriers, compared to controls, became evident in individuals born 1825 and after, following a sigmoid curve showing a continuous decrease that leveled out at the present average at the beginning of the 20th century (*P*≤3.3*10^−13^, Figure 3C).

**Table 1:** The effect of year of birth, fitted with a polynomial regression, and the geographical and genetic (maternal/paternal) origin on the lifespan of carriers.

	DF	MS	F	P
**Birth Year (3)**	3	6622.2	43.705	2.2*10^–16^
**Geographical origin**	1	1563.3	10.317	0.0016
**Genetic origin**	1	1242.1	8.197	0.0048
**Residuals**	145	151.5		

DF, MS, and F signify degrees of freedom, mean square and F-statistic, respectively.

**Figure 4:**
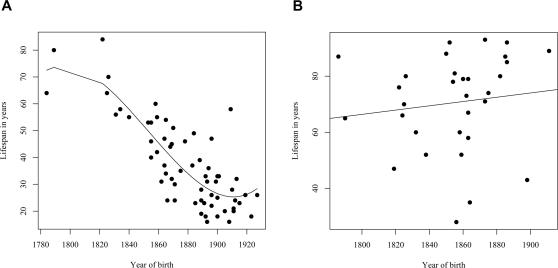
Families without DNA diagnosis. (A)The relationship between the life span of L68Q mutation carriers (n = 64; assumed carrier group) in families without DNA diagnosis and their year of birth between the years 1780 and 1920. The line presents the fitted relationship from a polynomial regression (*R^2^* = 0.68). (B) Relationship between the life span of 31 spouses of L68Q mutation carriers in families without DNA diagnosis and their year of birth. The line represents the fitted relationship from a linear regression (*b* = 0.076, *P*<0.494, *R^2^* = 0.02).

A similar reduction in life span was seen in a separate analysis of the assumed carrier group (Figure 4A), whose spouses (n = 31) had a slight but insignificant increase in life span (Figure 4B), thus supporting the notion that those six families were also *bona fide* HCCAA families. However, due to increasing early death of carriers in the 20^th^ century the L68Q mutation has now become extinct in these families and in five of the nine families where carrier status could be confirmed with genotyping.

### Parent-of-Origin Effect

**Figure 5:**
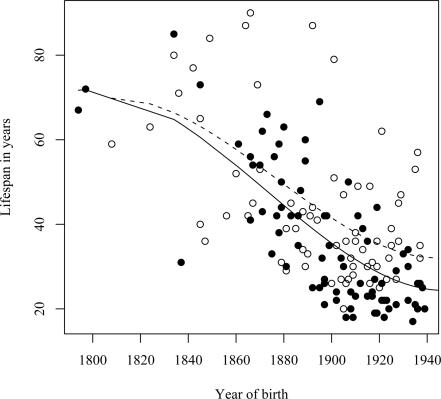
Relationship between the life span of L68Q mutation carriers and their year of birth from the year 1800 to 1940. In this case the data is categorized according to maternal/paternal inheritance. Individuals that inherited the mutant gene from their mother are shown as filled circles; individuals that inherited the gene from their father are shown as open circles. Fitted lines from polynomial regressions are shown for both categories. The dashed line shows the expected values for individuals that inherited the gene from their fathers, the solid line shows the expected values for individuals that inherited the gene from their mothers.

We observed a parent-of-origin effect on the life span of L68Q carriers (Table 1) that is most notable for people born after 1900 (Figure 5). The data indicate that carriers who inherited the L68Q mutation from their mother (n = 53; 29 sons and 24 daughters) lived 27 years on average (standard deviation (S.D.) = 7.78), whereas those who inherited the mutation from their father (n = 51; 23 sons and 28 daughters) lived 36.4 years on average (S.D. = 11.44). This 9.4 year difference in life span is highly significant (*t*-test, *P*≤0.001). It should be noted that of the handful of mutation carriers born after 1900 (1–2%) who achieved a “normal” life span [14], all inherited the mutation from their father.

### Geographic Difference in Life Span

**Figure 6:**
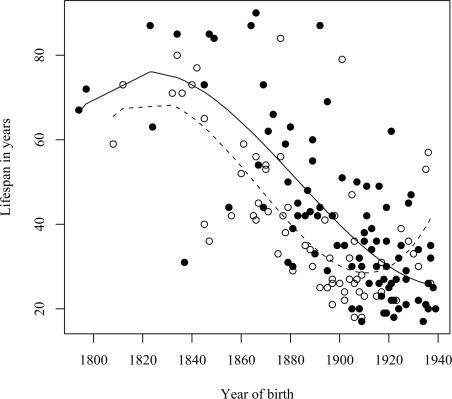
A comparison of the onset, and trend, of life span reduction between two geographic regions. Filled circles are individuals from the North-West of Iceland (region A in Figure 1), open circles are individuals from the West and South of Iceland (regions B and C in Figure 1). Fitted lines from polynomial regressions are shown for both categories. The dashed lines show the expected values for regions B and C combined, the solid line shows the expected values for region A. The difference between these lines indicates an approximately 20 year delay in the onset of life span reduction in the North-West of Iceland.

A significant difference (*P*≤0.0016) was seen in the timing of life span reduction between carriers from the remote Northwest coastal region A and those from the South and West regions B and C combined (Figures 1 and 6, and Table 1). The life span reduction in region A was delayed by about 20 years compared to the other regions.

## Discussion

In this study of HCCAA families with the cystatin C L68Q mutation we show that the life span of L68Q carriers in all the families underwent a reduction in the 19^th^ century reaching the present day average of 30 years around 1900. Concurrently a parent-of-origin effect became evident whereby those who inherited the disease gene from the mother had, on average, a 9.4 years shorter life span compared to those who inherited it from the father.

**Figure 7:**
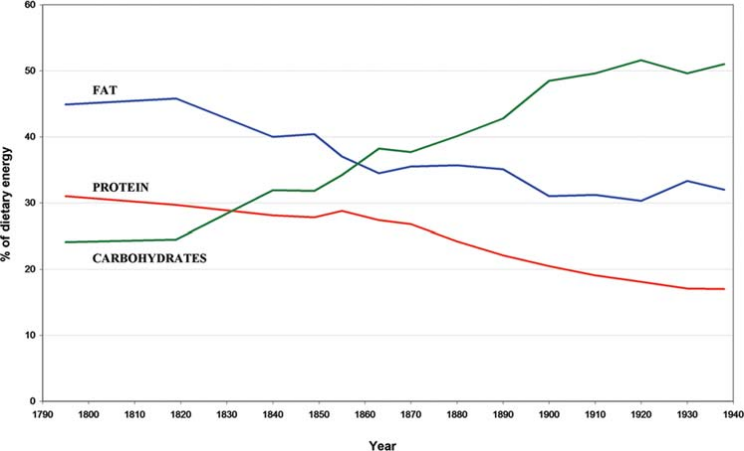
Changes in the percentage of dietary energy. Derived from fat (blue line), protein (red line), and carbohydrates (green line) in Iceland from the year 1790–1940 (adapted from Jonsson [29]). Iceland was an isolated island with relatively little importation of food at the beginning of our study period, which then underwent drastic changes of dietary habits during the latter part of the 19th century. Records of imported food items per capita show a shift in the proportion of energy derived from carbohydrates from 25 % in 1840 to approximately 50 % in 1900; the proportion of energy derived from fat and protein decreased accordingly.

How can we account for the rather drastic reduction in the life span of L68Q carriers that seems to have occurred three centuries after the mutation arose? It is unlikely that this is due to an additional, but currently undocumented, mutation acting as a modifier of the risk conferred by the L68Q mutation. Indeed, an additional mutation would need to have occurred many times in several different extended families. Alternatively, and even less plausible, a putative modifier mutation would have needed to spread from very low frequency to near fixation in each of the L68Q carrier families in the space of a few generations. Rather, we postulate the involvement of some environmental factor that came into play around 1825 and reached saturation around 1900, possibly a dietary factor such as the increased consumption of carbohydrates (Figure 7) or salt (for food preservation). The notion of an environmental modifier of disease risk conferred by the L68Q mutation is supported by the observation that the decrease in life span was delayed by about 20 years in the remote Northwest coastal region A compared to the West and South regions B and C combined. Thus, the life-style changes associated with economic development in Iceland during the 19^th^ century occurred later in remote regions than in regions closer to the capital Reykjavik.

It is interesting that in the same time period (the 19^th^ century) a parent-of-origin effect of the L68Q mutation is seen in the context of the life span reduction of carriers. This could be explained by a transgenerational epigenetic mechanism [15], such as the one described for the agouti viable yellow mutation in mice [16],[17] where the frequency of disease in offspring can be changed with dietary methyl supplementation of pregnant dams. However, although cystatin C has a large CpG region in the promoter, the gene is not known to be a metastable epiallele or reside in a known imprinting region.

The conclusion that environmental factors alter the phenotypic impact of mutations underlying serious heritable disorders is not novel. A classic example is the dietary treatment of phenylketouria (PKU) patients to prevent cognitive damage [18]. What is unusual about the findings reported here is rather that the detrimental phenotypic impact of the L68Q mutation appears to have emerged in reaction to life-style changes that fall within the normal range of behavior of a single population in the space of a few generations. Even this may not be without parallel, as a reduced life span over time coupled with a maternal effect has also been described in another familial amyloid disease in Sweden, i.e. FAP (familial amyloidotic polyneuropathy) which is caused by a mutation (V30M) in the transthyretin gene [19]. Such diseases challenge simplistic views of Mendelian diseases as solely genetic in nature and insensitive to environmental factors. An understanding of the phenotypic flexibility of the L68Q mutation might provide the possibility for preventive or therapeutic strategies to deal with HCCAA. Such an understanding may also be relevant to other common but pathophysiologically related disorders such as Alzheimer's disease, where cerebral amyloid angiopathy (CAA) is very common. Finally it may also present an opportunity for more general insights into the complex relationship between genotype and environment in human disease.

## Materials and Methods

### Family Data

A total of 36 individuals from nine families with known histories of ICH were diagnosed as carriers of the L68Q mutation through direct genotyping [6]. The ancestors of these individuals were found by tracing pedigrees, either with the help of Arnason [3], published pedigrees [20], or with the deCODE Genetics genealogy database that contains information about the relationships between 740.000 Icelanders, past and present [13]. Ancestors who were obligate carriers of L68Q were defined as parents or common ancestors of known carriers whose inferred carrier status minimizes the number of transmission events of the mutation in the genealogies. For most individuals, the only available phenotypic information was age at death, as the first signs of HCCAA can be subtle such as personality changes and dementia. Death certificates (first issued in Iceland in 1911) and parish records were also checked and the cause of death was noted when known. The year of birth, and death, of spouses of obligate L68Q carriers were noted when available.

The nine extended families thus defined contain 202 individuals, including 92 ancestors who are obligate carriers. Although the cause of death for these obligate carriers was often consistent with ICH, we also included those who died from other causes such as drowning or infections. In some cases, family members with no offspring or contemporary descendants were defined as obligate carriers, when their death certificate (n = 61) or their parish record (n = 17) indicated brain hemorrhage. Individuals were excluded if no information was available about cause of death. Limiting the analyses to all carriers born before 1940 (to exclude those who have an exceptionally long life span) we hereafter refer to 157 carriers as the *confirmed carrier group*.

In addition to the aforementioned known and obligate carriers, there are 34 deceased individuals who are strongly suspected of having been carriers of the L68Q mutation, but for whom no DNA samples were available. The inference of carrier status for these individuals is based on death certificates (n = 17) and parish records (n = 17) that indicate brain hemorrhage as the cause of death. Using the same approach as before to trace obligate carrier ancestors, we identified six new families and 30 new obligate carriers. We refer to this set of 64 individuals as the *assumed carrier group*.

### Age of the Mutation

In order to gain insight into the history of the cystatin C L68Q mutation in the Icelandic gene pool, we estimated its age by examining the decay of linkage disequilibrium (LD) between it and 20 surrounding microsatellites in the 36 known carriers and 722 non-carrier controls. The microsatellites spanned the physical map positions 14.7–38.3 Mb (NCBI build 35) on chromosome 20, with the L68Q mutation located at position 23,563,968 (see Table S1).

PCR amplifications were set up, run, and pooled on Gilson Cyberlab robots. The reaction volume was 5 µl, and, for each PCR, 20 ng of genomic DNA was amplified in the presence of 2 pmol of each primer, 0.25 U AmpliTaq Gold, 0.2 mM dNTPs, and 2.5 mM MgCl_2_ (buffer was supplied by the manufacturer, Applera). Cycling conditions were as follows: 95°C for 10 min, followed by 37 cycles of 94°C for 15 s, annealing for 30 s at 55°C, and 1 min extension at 72°C. One primer of each primer pair was fluorescently labeled. The PCR products were pooled into panels of 8–16 markers, mixed with size standards, and analyzed on ABI 3700 sequencing machines using Genescan (version 3.0) peak-calling software (Applied Biosystems, Foster City, CA). Alleles were automatically called using DAC, an allele-calling program developed at deCODE Genetics [21], and the program DecodeGT was used to fractionate called genotypes, according to quality, and to edit when necessary [22].

First, we established the microsatellite allele states of the chromosome on which the L68Q mutation is most likely to have occurred, referred to hereafter as the founder alleles. This was achieved by picking the microsatellite allele that exhibited the strongest LD (measured by D') with the cystatin C mutation and showed the greatest difference in frequency between the mutation carriers and controls. Haplotype frequencies were estimated using the expectation maximization (EM) algorithm [23]. Age estimates of the mutation were then obtained based on the pattern of LD with each individual microsatellite based on the formula:
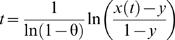
where *t* is the age of the mutation in generations, θ is the recombination rate in Morgans between the mutation and the flanking microsatellite, *x(t)* is the frequency of chromosomes carrying both the cystatin C mutation and the microsatellite ancestral allele and *y* is the frequency of chromosomes that do not carry the cystatin C mutation but do carry the microsatellite ancestral allele [24]. Age estimates based on individual microsatellites were also calculated using a correction, based on the Luria-Delbrück model, that takes into account the impact of population growth [25]. The corrected age is defined as:
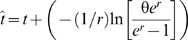
using the same notation as before with additional parameters, *t*, the time estimate based on the previous formula, and *r*, the rate of past exponential population growth per generation. Based on detailed information about the size of the Icelandic population during the past 250 years [26], we estimated *r* to have been 0.3. The recombination rates between loci were obtained by interpolating positions from the high-resolution recombination map estimated from the phase II HapMap project data, using physical map locations as the common frame of reference (www.hapmap.org) [27].

As age estimates based on individual loci can be subject to considerable measurement error, and are unreliable between alleles that are still in very strong LD, we also employed the method implemented in the DMLE+ software version 2.3 [28]. Using this approach we were able to use the LD pattern of all 20 microsatellites simultaneously with the L68Q mutation to provide a single age estimate. The DMLE+ software was run using 100,000 burn-in iterations and 100,000 calculation iterations, with *r* = 0.3.

### Web Resources

The URLs for data presented herein are as follows: Online Mendelian Inheritance in Man (OMIM), http://www.ncbi.nlm.nih.gov/sites/omim.

## Supporting Information

Table S1 Microsatellites used to estimate the age of the L68Q mutation in the cystatin C gene.
(0.07 MB DOC)
