# Supplementary material for: A Drastic Reduction in the Life Span of Cystatin C L68Q Carriers Due to Life-Style Changes during the Last Two Centuries
Source: PLoS Genet. 2008 Jun 20;4(6):e1000099. doi: 10.1371/journal.pgen.1000099 (PMC2409978; doi:10.1371/journal.pgen.1000099)
Supplement: Table S1 — Microsatellites used to estimate the age of the L68Q mutation in the cystatin C gene. (0.07 MB DOC) [file pgen.1000099.s001.doc]

**Supplementary Table 1. Microsatellites used to estimate the age of the L68Q mutation in the cystatin C gene**

|  |  |  |  | Carriers | Controls | | | Inferred SG20S1398A founder chromosome allele | | | LD and mutation age estimates for individual microsatellites | | |
| --- | --- | --- | --- | --- | --- | --- | --- | --- | --- | --- | --- | --- | --- |
| Locus | Physical position on chr 20 (NCBI build 35) | Physical distance from SG20S1398 (kb) | Genetic distance from SG20S1398 (Morgans) | 2N | 2N | No. of alleles | Expected Heterozygosity | Allele | Frequency in carriers | Frequency in controls | D' with SG20S1398 A (combined sample) | Mutation age (gens) | Luria & Delbruck corrected age (gens) |
| DG20S419 | 14714802 | 8849.166 | 0.142531415 | 70 | 650 | 10 | 0.7248 | 4 | 0.44444 | 0.33538 | 0.2413 | 8.998 | 10.991 |
| D20S98 | 15590917 | 7973.051 | 0.122014646 | 72 | 548 | 10 | 0.7729 | -8 | 0.18056 | 0.01095 | 0.65005 | 8.137 | 10.648 |
| D20S875 | 16590263 | 6973.705 | 0.101993722 | 72 | 708 | 10 | 0.8273 | -4 | 0.20833 | 0.14407 | 0.17287 | 15.950 | 19.059 |
| D20S605 | 17664743 | 5899.225 | 0.081474384 | 72 | 542 | 6 | 0.6825 | 0 | 0.66667 | 0.46125 | 0.71415 | 3.759 | 7.617 |
| DG20S592 | 18586696 | 4977.272 | 0.066500771 | 68 | 704 | 9 | 0.7787 | 0 | 0.375 | 0.20739 | 0.32978 | 15.679 | 20.214 |
| DG20S425 | 19594398 | 3969.57 | 0.047841344 | 44 | 680 | 8 | 0.7153 | 2 | 0.47222 | 0.19706 | 0.64514 | 8.716 | 14.348 |
| DG20S578 | 21604603 | 1959.365 | 0.01915527 | 72 | 658 | 9 | 0.3796 | 2 | 0.88889 | 0.77356 | 0.99964 | 0.018 | 8.701 |
| DG20S621 | 22672391 | 891.577 | 0.010288325 | 68 | 712 | 9 | 0.739 | 2 | 0.55556 | 0.16713 | 0.9552 | 4.241 | 14.996 |
| DG2S593 | 23028352 | 535.616 | 0.004895101 | 72 | 502 | 5 | 0.3364 | 0 | 0.875 | 0.80279 | 0.57202 | 108.207 | 121.438 |
| D20S871 | 23283572 | 280.396 | 0.001501083 | 68 | 370 | 16 | 0.8727 | 12 | 0.59722 | 0.13514 | 1 | 0.002 | 17.173 |
| SG20S1398 | 23563968 | 0 | 0 | 72 | 710 | 2 | 0 | A | 0.5 | 0 |  |  |  |
| D20S848 | 24281808 | 717.84 | 0.00790314 | 64 | 718 | 5 | 0.7429 | 2 | 0.51389 | 0.19916 | 0.99997 | 0.003 | 11.637 |
| DG20S911 | 24363784 | 799.816 | 0.008293906 | 72 | 536 | 10 | 0.7522 | 0 | 0.56944 | 0.28731 | 0.99997 | 0.004 | 11.477 |
| D20S65 | 25044710 | 1480.742 | 0.016840125 | 72 | 694 | 8 | 0.6097 | 0 | 0.73611 | 0.59078 | 0.67343 | 22.356 | 31.469 |
| DG20S917 | 25571268 | 2007.3 | 0.017776259 | 64 | 704 | 7 | 0.7462 | 0 | 0.59722 | 0.20597 | 0.99998 | 0.001 | 8.933 |
| D20S191 | 26105005 | 2541.037 | 0.018362742 | 72 | 708 | 7 | 0.6867 | -2 | 0.63889 | 0.34181 | 0.99998 | 0.001 | 8.825 |
| D20S425 | 29930472 | 6366.504 | 0.01972747 | 72 | 702 | 5 | 0.71 | 2 | 0.69444 | 0.30627 | 0.94326 | 2.800 | 11.385 |
| D20S878 | 31919789 | 8355.821 | 0.034228196 | 72 | 710 | 8 | 0.586 | -4 | 0.45833 | 0.17746 | 0.74201 | 8.220 | 14.968 |
| D20S847 | 34306862 | 10742.894 | 0.039626441 | 72 | 368 | 12 | 0.8509 | -12 | 0.34722 | 0.125 | 0.43378 | 19.461 | 25.722 |
| D20S859 | 35866837 | 12302.869 | 0.050493932 | 72 | 716 | 7 | 0.7263 | 8 | 0.38889 | 0.28771 | 0.20921 | 29.474 | 34.926 |
| D20S107 | 38305970 | 14742.002 | 0.079580888 | 72 | 706 | 10 | 0.7755 | 6 | 0.33333 | 0.25637 | 0.1567 | 21.865 | 25.801 |
